# Supplementary material for: Risk factors and risk profiles for neck pain in young adults: Prospective analyses from adolescence to young adulthood—The North-Trøndelag Health Study
Source: PLoS One. 2021 Aug 12;16(8):e0256006. doi: 10.1371/journal.pone.0256006 (PMC8360564; doi:10.1371/journal.pone.0256006)
Supplement: S2 Table — (DOCX) [file pone.0256006.s002.docx]

**S2 Table. Univariate analyses of the association between potential risk factors in adolescence and persistent neck pain in young adulthood**

|  | **Sample I** | **Sample II** |
| --- | --- | --- |
| **Variables** | **Association with persistent neck pain (OR and 95% CI)** | **Association with persistent neck pain (OR and 95% CI)** |
| Sex  Male  female | 1  2.3 (1.7-3.2)* | 1  2.3 (1.4-3.7)* |
| BMI  Normal weight  Overweight/obese | 1  1.3 (0.9-1.8)* | 1  1.0 (0.6-1.7) |
| Family economy  Average  Better  Worse | 1  0.9 (0.6-1.4)  1.4 (0.9-2.3)* | 1  1.0 (0.5-1.9)  2.9 (1.6-5.6)* |
| Headache/migraine  seldom  Often^¥^ | 1  2.8 (2.1-3.8)* | 1  2.2 (1.3-3.7)* |
| Neck/shoulder pain  Seldom  Often^¥^ | 1  3.3 (2.5-4.5)* |  |
| Back pain  Seldom  Often^¥^ | 1   - 1. (1.9-3.6)* | 1  1.0 (0.4-2.4) |
| Abdominal pain  Seldom  often^¥^ | 1  2.0 (1.4-2.9)* | 1  1.6 (0.8-3.2) |
| Number of pain sites  0  1  2  3 or more | 1  1.4 (0.9-2.1)  2.0 (1.2-3.2)*  4.7 (3.3-6.8)* | 1  1.2 (0.6-2.1)  1.1 (0.5-2.7)  3.1 (1.1-8.3)* |
| Physical activity  High level  Moderate level  Low level | 0.9 (0.6-1.3)  1  1.7 (1.2-2.3)* | 0.9 (0.5-1.6)  1  1.6 (0.9-2.8)* |
| Difficulty falling asleep  Never  Sometimes  Often | 1  1.5 (1.1-2.0)  2.1 (1.4-3.1)* | 1  1.1 (0.7-1.7)  1.7 (0.9-3.3)* |
| Psychological distress^⸸^  <2.0  ≥2.0 | 1  2.1 (1.5-2.9)* | 1  1.7 (0.9-3.2)* |
| Self-esteem scale^⸷^ | 0.8 (0.8-0.9)* | 0.8 (0.7-0.9)* |
| Resilience^§^  Social competence  Family cohesion | 0.9 (0.9-1.0)  0.9 (0.9-0.9) * | 0.9 (0.9-1.0)  0.9 (0.9-1.0) |
| Loneliness  Seldom  Sometimes  Often/very often | 1  1.6 (1.2-2.2)*   - 1. (1.8-4.2)* | 1  1.4 (0.8-2.4)  2.9 (1.4-6.2)* |
| Sample I= all participants, Sample II= individuals at risk  *Variables with a significance level <0.1  ^¥^ Pain at least once per week during the last three months not related to any known disease or injury  ^⸷^ Rosenberg self-esteem scale, ^⸸^ Symptom check list, ^§^Resilience scale for adolescents | | |
