# Supplementary material for: Risk factors and risk profiles for neck pain in young adults: Prospective analyses from adolescence to young adulthood—The North-Trøndelag Health Study
Source: PLoS One. 2021 Aug 12;16(8):e0256006. doi: 10.1371/journal.pone.0256006 (PMC8360564; doi:10.1371/journal.pone.0256006)
Supplement: S3 Table — (DOCX) [file pone.0256006.s003.docx]

**S3 Table. Analyses of baseline characteristics of study participants and participants lost to follow-up**

|  | **Sample I** | | | **Sample II** | | |
| --- | --- | --- | --- | --- | --- | --- |
| **Characteristics at baseline** | **Follow-up responders**  **N= 1433** | **Lost to follow-up**  **N= 5852** | **p-value** | **Follow-up responders**  **N= 832** | **Lost to follow-up**  **N= 3565** | **p-value** |
| Age (*yr) mean*, *(SD)* | 15.9 (1.8) | 15.8 (1.7) | 0.17 | 15.8 (1.7) | 15.7 (1.7) | 0.11 |
| Sex, *n (%)*  Female  Male | 912 (63.6)  521 (36.4) | 2786 (47.6)  3066 (52.4) | <0.00* | 479 (57.6)  353 (42.4) | 1466 (41.1)  2099 (58.9) | <0.00* |
| Family economy, *n (%)*  Better  Average  Worse  *Missing* | 215 (15.0)  1006 (70.2)  118 (8.2)  94 (6.6) | 3887 (17.7)  1037 (66.4)  522 (8.9)  406 (6.9) | 0.01* | 127 (15.3)  598 (71.9)  59 (7.1)  48 (5.8) | 634 (17.8)  2457 (68.9)  245 (6.9)  229 (6.4) | 0.19 |
| BMI *(kg/m^2^), n (%)*  Normal  Overweight/obese  *Missing* | 1037 (72.4)  315 (22.0)  81 (5.7) | 4076 (69.7)  1309 (22.4)  467 (8.0) | 0.43 | 621 (74.6)  161 (19.4)  50 (6.0) | 2542 (71.3)  765 (21.5)  258 (7.2) | 0.12 |
| School type, *n (%)*  Middle school students, *n*  High school students, *n*  *Missing* | 808 (56.4)  592 (41.3)  33 (2.3) | 3411 (58.3)  2373 (40.6)  68 (1.2) | 0.39 | 489 (58.8)  326 (39.2)  17 (2.0) | 2221 (62.3)  1308 (36.7)  36 (1.0) | 0.11 |
| Subjective health, *n (%)*  Good  Poor  *Missing* | 1283 (89.5)  137 (9.6)  13 (0.9) | 5133 (87.7)  629 (10.7)  90 (1.5) | 0.16 | 783 (94.1)  44 (5.3)  5 (0.6) | 3295 (92.4)  234 (6.6)  36 (1.0) | 0.16 |
| Neck/shoulder pain, *n (%)*  Often^¥^  *Missing* | 259 (18.1)  25 (1.7) | 998 (17.1)  182 (3.2) | 0.49 |  |  |  |
| Headache/migraine, *n (%)*  Often^¥^  *Missing* | 339 (23.7)  26 (1.8) | 1256 (21.5)  123 (2.1) | 0.08 | 118 (14.2)  6 (0.7) | 458 (12.8)  14 (0.4) | 0.28 |
| Back pain, *n (%)*  Often^¥^  *Missing* | 266 (18.6)  40 (2.8) | 999 (17.1)  199 (3.4) | 0.21 | 58 (7.0)  10 (1.2) | 246 (6.9)  22 (0.6) | 0.90 |
| Number of pain sites, *n (%)*  0  1  2  3 or more  *Missing* | 668 (46.6)  237 (16.5)  142 (9.9)  213 (14.9)  173 (12.1) | 2852 (48.7)  936 (16.0)  530 (9.1)  754 (12.9)  780 (13.3) | 0.14 | 536 (64.4)  142 (17.1)  57 (6.9)  22 (2.6)  75 (9.0) | 2386 (66.9)  531 (14.9)  222 (6.2)  127 (3.6)  229 (8.4) | 0.18 |
| Physical activity level, *n (%)*  High level  Moderate level  Low level  *Missing* | 526 (36.7)  513 (35.8)  372 (26.0)  22 (1.5) | 2368 (40.5)  2006 (34.3)  1388 (23.7)  90 (1.5) | 0.02* | 329 (39.5)  299 (35.9)  190 (22.8)  14 (1.7) | 1545 (43.3)  1216 (34.1)  773 (21.7)  31 (0.9) | 0.19 |
| Psychological distress^⸸^, *n (%)*  <2.00  ≥2.00  *Missing* | 1124 (78.4)  266 (18.6)  43 (3.0) | 4559 (77.9)  1069 (18.3)  224 (3.8) | 0.90 | 730 (87.7)  80 (9.6)  22 (2.6) | 3100 (87.0)  367 (10.3)  98 (2.7) | 0.55 |
| Loneliness, *n (%)*  Often  Sometimes  Seldom  *Missing* | 119 (8.3)  319 (22.3)  899 (62.7)  96 (6.7) | 530 (9.1)  1305 (22.3)  3625 (61.9)  392 (6.7) | 0.65 | 41 (4.9)  152 (18.3)  586 (70.4)  53 (6.4) | 194 (5.4)  649 (18.2)  2502 (70.2)  220 (6.2) | 0.84 |
| Self-esteem^⸷^, *mean (SD)*  *Missing* | 12.0 (4-16)  77 (5.4) | 12.2 (4-16)  360 (6.2) | <0.00^§^* | 12.0 (4-16)  42 (5.0) | 13.0 (4-16)  178 (5.0) | <0.00^§^* |
| Sample I= all participants, Sample II= individuals at risk  *Statistically significant differences between included participants and participants lost to follow-up  ^¥^ Pain at least once per week during the last three months not related to any known disease or injury  ^⸸^ Symptom check list (1-4), ^⸷^ Rosenberg self-esteem scale (0-12), ^§^ Mann Whitney U test | | | | | | |
