# Supplementary figures and images for: Risk factors and risk profiles for neck pain in young adults: Prospective analyses from adolescence to young adulthood—The North-Trøndelag Health Study
Source: PLoS One. 2021 Aug 12;16(8):e0256006. doi: 10.1371/journal.pone.0256006 (PMC8360564; doi:10.1371/journal.pone.0256006)

**
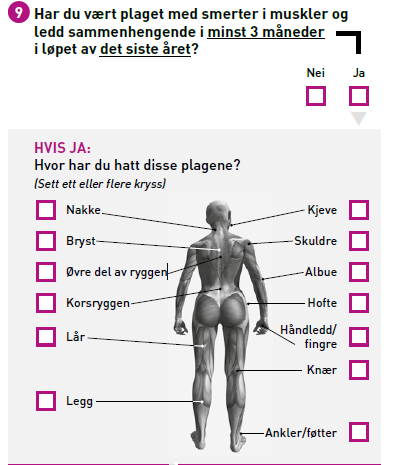
Outcome measure from HUNT4 in the original language (Norwegian)**

Supplement: S1 Questionnaire — (DOCX) [file pone.0256006.s004.docx]
