# Supplementary material for: Risk factors and risk profiles for neck pain in young adults: Prospective analyses from adolescence to young adulthood—The North-Trøndelag Health Study
Source: PLoS One. 2021 Aug 12;16(8):e0256006. doi: 10.1371/journal.pone.0256006 (PMC8360564; doi:10.1371/journal.pone.0256006)
Supplement: S2 Questionnaire — (DOCX) [file pone.0256006.s005.docx]

**Questions from HUNT4- outcome measure**

*(This question is translated to English only to be transparent of the methods used and is not validated for research)*

- In the last year, have you had pain or stiffness in muscles or joints that has lasted at least 3 consecutive months?

□ Yes □ No

- If Yes, Where have you had this pain or stiffness?


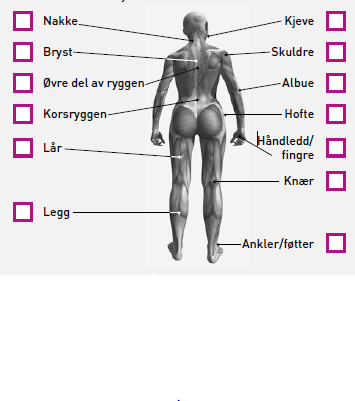


Neck

Chest

Upper back

Lower back

Thigh

shin

Jaw

Shoulders

Elbow

Hip

Wrist/ fingers

Knees

Ankles/ feet
