# Supplementary material for: Risk factors and risk profiles for neck pain in young adults: Prospective analyses from adolescence to young adulthood—The North-Trøndelag Health Study
Source: PLoS One. 2021 Aug 12;16(8):e0256006. doi: 10.1371/journal.pone.0256006 (PMC8360564; doi:10.1371/journal.pone.0256006)
Supplement: S3 Questionnaire — (PDF) [file pone.0256006.s006.pdf]

UNG hunt 3

Helseundersøkelsen i Nord-Trøndelag

Spørreskjema for ungdom

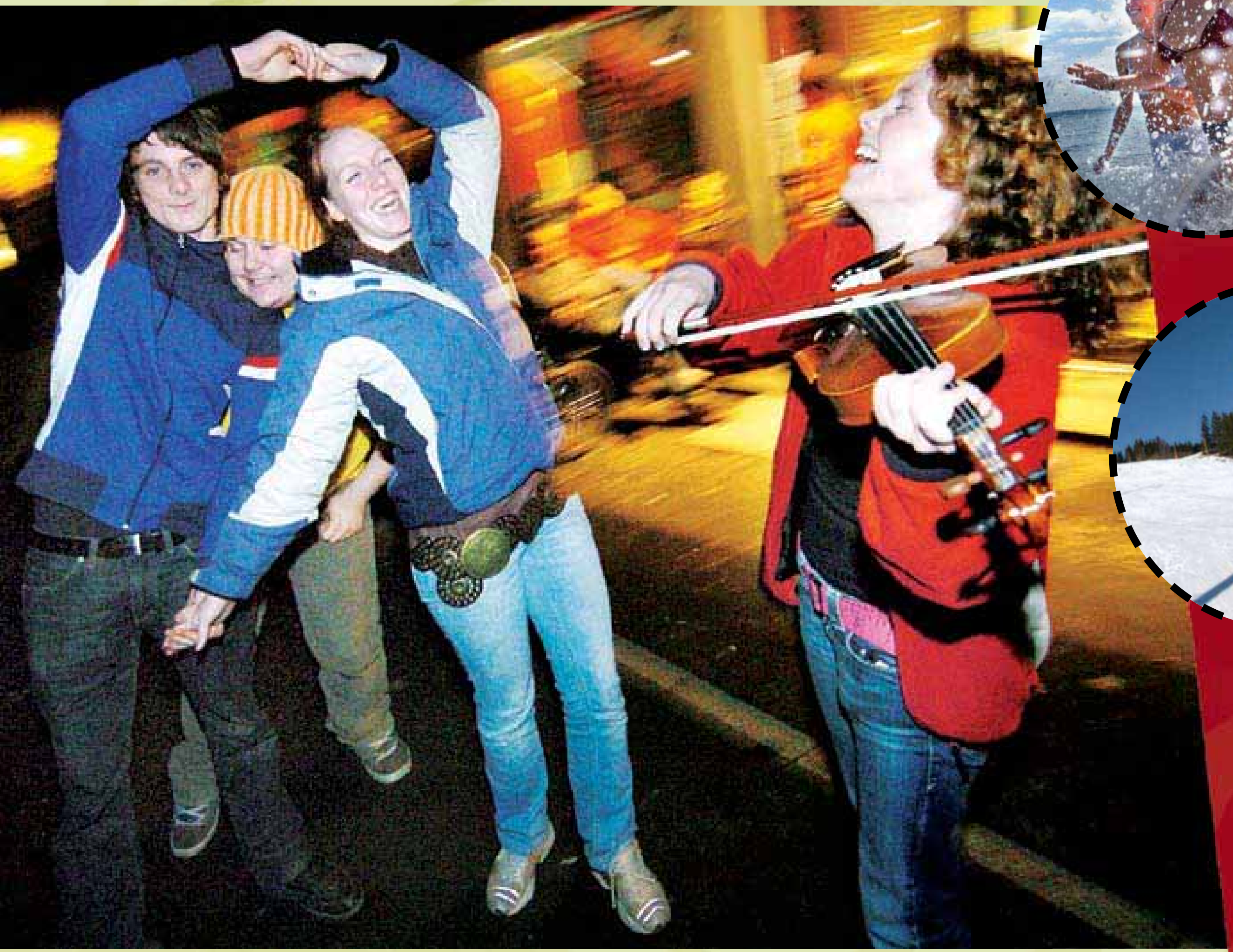

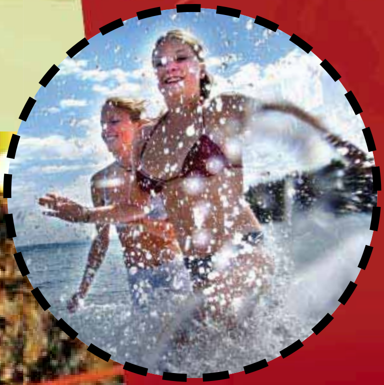

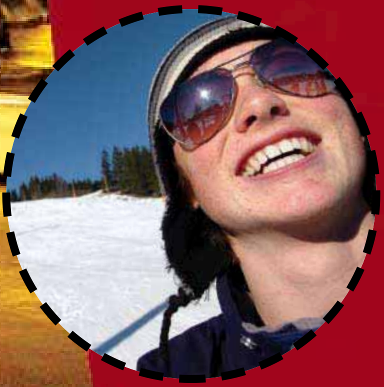

Nå er det din tur til å delta i den store helseundersøkelsen i Nord-Trøndelag!

Vi håper du har lest igjennom informasjonsbrosjyren du fikk om Ung-HUNT og bestemt deg for å være med!

92300000019

NTNU
Det skapende universitet

En time for bedre helse

Samtykkeerklæringen ligger sammen med spørreskjemaet. Sjekk at det er ditt navn som står der! Kryss av for om du vil delta eller ikke, og undertegn. Lever samtykket til læreren.

Navnet ditt skal IKKE være med på spørreskjemaet!

Sett et kryss i rutene ☒ du synes passer for deg. Svar så godt du kan! Spørsmål du ikke ønsker å svare på, kan du hoppe over.

Når du er ferdig, legger du spørreskjemaet i den konvolutten du har fått, klistrer igjen og leverer konvolutten til læreren. Lever spørreskjemaet selv om du ikke har svart på alle spørsmålene.

Alle svarene dine blir behandlet med taushetsplikt!

Ingen på skolen får se svarene dine.

Hvis du ønsker å snakke med noen om undersøkelsen, kan du ta kontakt med Ung-HUNT-sykepleieren når hun kommer på skolen din, eller ringe HUNT forskningssenter i Verdal (se baksiden).

Lykke til og tusen takk!

Vennlig hilsen

Turid Lingaas Holmen
Førsteamanuensis, barnelege
Leder for Ung-HUNT

Jostein Holmen
Professor, daglig leder
HUNT forskningssenter

Stig A. Slørdahl
Professor, dekanus
Det medisinske fakultet, NTNU

Slik fyller du ut skjemaet

- Skjemaet vil bli lest av en maskin
- Det er derfor viktig at du krysser slik: Rett ☒ Galt ☑ ☐✗
- Krysser du feil sted, tar du bort krysset ved å fylle boksen slik: ■
- Skriv tydelige tall: 1 2 3 4 5 6 7 8 9 0
- Bruk bare svart eller blå penn. Ikke bruk blyant eller tusj.

Dato for utfylling av skjema ☐ / ☐ 20 ☐
DATO MÅNED ÅR

1. For de som går i ungdomsskolen: **Hvilken linje vil du velge på videregående skole?**

Allmennfag (studiekompetanse) ☐ Yrkesfag ☐ Vet ikke ☐

2. **Hvilke planer for videre utdanning har du?** *Sett ett eller flere kryss*

- Høgskole eller universitet i 4 år eller mer ☐
- Høgskole eller universitet mindre enn 4 år ☐
- Annen yrkesutdanning ☐
- Ingen planer ☐
- Vet ikke ☐

OM DER DU BOR

3. **Hvilken type bolig (hus) bor du i?** *Sett bare ett kryss*

- Enebolig ☐
- Rekkehus/2-4 mannsbolig ☐
- Blokk/leilighet ☐
- Gårdsbruk med dyrehold ☐
- Gårdsbruk uten dyrehold ☐
- Annen bolig ☐

4. **Hvem bor du sammen med nå?** *Sett ett eller flere kryss*

- Mor ☐
- Far ☐
- 1-2 søsken ☐
- 3 eller flere søsken ☐
- Mors nye mann eller samboer ☐
- Fars nye kone eller samboer ☐
- Fosterforeldre ☐
- Adoptivforeldre ☐
- Besteforeldre/andre ☐
- Ektefelle/samboer ☐
- Venner ☐
- Alene/på hybel ☐

5. **Hvis mor og far ikke bor sammen, hvem bor du sammen med?**

Mest med mor ☐ Mest med far ☐ Like mye hos begge ☐

6. **Er det kjæledyr i boligen hjemme hos deg?**

- Nei ☐
- Ja, katt ☐
- Ja, hund ☐
- Ja, andre pelskledde dyr ☐
- Ja, fugl ☐
- Ja, andre ☐

92300000019

OM HELSA DI

7. Hvordan er helsa di nå? *Sett ett kryss*

- Dårlig ☐
- Ikke helt god ☐
- God ☐
- Svært god ☐

8. Er du hemmet på noen av disse måtene? *Sett ett kryss for hver linje*

	Nei	Litt	Middels	Mye
• Er bevegelseshemmet	☐	☐	☐	☐
• Har nedsatt syn	☐	☐	☐	☐
• Har nedsatt hørsel	☐	☐	☐	☐
• Hemmet pga. kroppslig sykdom	☐	☐	☐	☐
• Hemmet pga. psykiske plager	☐	☐	☐	☐

9. Hvor plaget har du vært <u>de siste 12 månedene</u> med noe av det som står nedenfor: *Sett ett kryss for hver linje*

	Ikke noe	Litt	Mye
• Hjertebank	☐	☐	☐
• Treg mage	☐	☐	☐
• Diaré/løs mage	☐	☐	☐
• Vekslende treg mage og diaré	☐	☐	☐
• Oppblåsthet	☐	☐	☐
• Kvalme	☐	☐	☐

OM ALLERGI

10. Er du allergisk? Ja ☐ Nei ☐ Vet ikke ☐

Hvis <u>ja</u>, **hva kjenner du selv at du er allergisk for?** *Sett ett eller flere kryss*

- Gress/trær ☐
- Husstøv ☐
- Hund ☐
- Katt ☐
- Hest ☐
- Mat ☐
- Annet ☐
- Vet ikke ☐

11. Har du tatt allergitest hos lege (blodprøve, hudtest)**?** Ja ☐ Nei ☐ Vet ikke ☐

Hvis <u>ja</u>, **hva reagerte du allergisk på i testen?** *Sett ett eller flere kryss*

- Ingenting ☐
- Gress/trær ☐
- Husstøv ☐
- Hund ☐
- Katt ☐
- Hest ☐
- Mat ☐
- Annet ☐
- Vet ikke ☐

OM LUFTVEISPLAGER

12. Har du noen gang hatt tung pust eller piping/surkling/tetthet i brystet?

Ja ☐ Nei ☐

HVIS DU HAR SVART «**NEI**» GÅ TIL SPØRSMÅL **15**

13. Har du hatt tung pust eller piping/surkling/tetthet i brystet i løpet av de siste 12 månedene ?

Ja ☐ Nei ☐

HVIS DU HAR SVART «**NEI**» GÅ TIL SPØRSMÅL **15**

14. Hvor mange anfall med tung pust eller piping/surkling/tetthet i brystet har du hatt i løpet av de siste 12 månedene?

Ingen ☐ 1 til 3 ☐ 4 til 12 ☐ Mer enn 12 ☐

---

15. Har du, eller har du hatt astma?

Ja ☐ Nei ☐

Hvis ja, har lege sagt at du har/har hatt astma?

Ja ☐ Nei ☐

16. Har du i løpet av de siste 12 månedene hatt tung pust eller piping/surkling/tetthet i brystet under eller etter fysisk trening, aktiv lek eller mosjonering?

Ja ☐ Nei ☐

17. Har du i løpet av de siste 12 månedene hatt tørr hoste om natten uten å være forkjølet eller ha annen luftveisinfeksjon?

Ja ☐ Nei ☐

OM NESEPLAGER

18. Har du i løpet av de siste 12 månedene hatt problemer med nysing, rennende eller tett nese uten å ha vært forkjølet eller å ha hatt influensa?

Ja ☐ Nei ☐

HVIS DU HAR SVART «**NEI**» GÅ TIL SPØRSMÅL **21**

19. Har disse neseproblemene vært ledsaget av kløende, rennende øyne?

Ja ☐ Nei ☐

20. Hvor mye har disse neseproblemene virket inn på den daglige aktiviteten din? *Sett ett kryss*

Ikke i det hele tatt ☐ Litt ☐ Mye ☐ Veldig mye ☐

---

21. Har du noen gang hatt høysnue eller neseallergi?

Ja ☐ Nei ☐

OM UTSLETT

22. **Har du hatt kløende utslett i løpet av de siste 12 månedene?** Ja ☐ Nei ☐

HVIS DU HAR SVART «**NEI**» GÅ TIL SPØRSMÅL **25**

23. **Har dette kløende utslettet sittet på noen av de følgende stedene: albuebøyene** (på innsiden), **bak knærne, foran på anklene, under baken eller rundt hals, ører eller øyne?** Ja ☐ Nei ☐

24. **Hvor ofte i gjennomsnitt har du blitt holdt våken om natten på grunn av dette kløende utslettet?** *Sett ett kryss*

Ingen ganger ☐ Mindre enn en natt per uke ☐ En natt eller mer per uke ☐

25. **Har du noen gang hatt eksem?** Ja ☐ Nei ☐

Hvis ja, **har lege sagt at du har eller har hatt "atopisk eksem"?** Ja ☐ Nei ☐

OM KVISER

26. **Har du noen gang vært plaget med kviser?** Ja ☐ Nei ☐

HVIS DU HAR SVART «**NEI**» GÅ TIL SPØRSMÅL **31**

27. **Hvor sitter/satt kvisene dine?** *Sett ett eller flere kryss*

- Panne .... ☐
- Kinn .... ☐
- Skuldre .... ☐
- Andre steder .... ☐
- Nese .... ☐
- Bryst .... ☐
- Rygg .... ☐

28. **Hvor mye har kvisene plaget deg?** *Sett ett kryss* Svært mye ☐ Mye ☐ Litt ☐ Ikke noe ☐

29. **Har du brukt reseptfrie kremer, hudvann el. lign mot kviser?**
(F.eks. kjøpt på apotek eller parfymeri, ikke foreskrevet av lege) Ja ☐ Nei ☐

Hvis ja, **har denne behandlingen hjulpet?** *Sett ett kryss* Nei ☐ Noe ☐ Godt ☐

30. **Har du oppsøkt lege på grunn av kviser?** Ja ☐ Nei ☐

Hvis ja, **har du fått noen av disse behandlingene av legen?** *Sett ett kryss for hver linje*

	Ja	Nei
• **Lokalbehandling** (f.eks. kremer eller oppløsninger)	☐	☐
• **Antibiotika-tabletter** (tetracykliner)	☐	☐
• **Roaccutan tabletter**	☐	☐

Hvis ja, **har denne behandlingen hjulpet?** *Sett ett kryss* Nei ☐ Noe ☐ Godt ☐

OM SMERTER

31. Hvor ofte har du hatt noen av disse plagene i løpet av <u>de siste 3 månedene</u>?
(Uten at du har skadet deg eller har en kjent sykdom som er årsak til smertene)
Se på figurene og sett ett kryss for hver linje

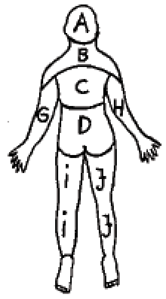

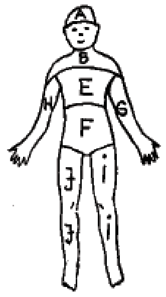

	Aldri/ sjelden	Omtrent en gang i måneden	Omtrent en gang i uka	Flere ganger i uka	Nesten hver dag
A. Hodepine/migrene	☐	☐	☐	☐	☐
B. Nakke-/skuldersmerter	☐	☐	☐	☐	☐
C. Smerter i øvre del av ryggen	☐	☐	☐	☐	☐
D. Smerter i nedre del av ryggen/setet	☐	☐	☐	☐	☐
E. Smerter i brystkassen	☐	☐	☐	☐	☐
F. Magesmerter	☐	☐	☐	☐	☐
G. Smerter i venstre arm	☐	☐	☐	☐	☐
H. Smerter i høyre arm	☐	☐	☐	☐	☐
I. Smerter i venstre bein	☐	☐	☐	☐	☐
J. Smerter i høyre bein	☐	☐	☐	☐	☐
Andre smerter	☐	☐	☐	☐	☐

HVIS DU HAR SVART **«ALDRI/SJELDEN»** PÅ ALT: GÅ TIL SPØRSMÅL **34**

Hvis du har hatt smerter i løpet av de siste 3 månedene:

32. Stemmer noe av det som står nedenfor på deg? *Sett ett kryss for hver linje*

	Stemmer	Stemmer ikke
• Smerter gjør det vanskelig for meg å sovne	☐	☐
• Smerter forstyrrer den gode nattesøvnen min	☐	☐
• Smerter gjør det vanskelig å sitte i skoletimen	☐	☐
• Smerter gjør det vanskelig for meg å gå mer enn en kilometer	☐	☐
• På grunn av smerter har jeg problemer i gymtimen	☐	☐

33. Har smertene alt i alt hindret deg i å utføre daglige aktiviteter? *Sett ett kryss for hver linje*

	Nei	Ja, av og til	Ja, ofte
• På skolen	☐	☐	☐
• I fritida	☐	☐	☐

Hvis <u>ja</u>, **hva slags smerter hindret deg i å utføre daglige aktiviteter?** *Sett ett eller flere kryss*

Hodepine/migrene ☐ Magesmerter ☐ Muskel-/leddsmerter ☐ Andre smerter ☐

92300000019

OM ANDRE SYKDOMMER

34. Har lege sagt at du har: *Sett ett kryss for hver linje*

	Ja	Nei
• Epilepsi	☐	☐
• Diabetes (sukkersyke)	☐	☐
• Migrene	☐	☐
• Barneleddgikt	☐	☐
• Andre sykdommer som har vart over 3 måneder	☐	☐

OM MEDISINER

35. Hvor ofte i de siste 3 månedene har du brukt reseptfrie medisiner mot noen av plagene nedenfor? (medisiner ikke foreskrevet av lege, f.eks. kjøpt på butikk eller apotek)

Sett ett kryss for hver linje

	Aldri	1 dag i uka eller sjeldnere	2 dager i uka	3 dager i uka	4 dager i uka eller mer
• Hodepine/migrene	☐	☐	☐	☐	☐
• Muskel-/leddsmerter	☐	☐	☐	☐	☐
• Ryggsmerter	☐	☐	☐	☐	☐
• Magesmerter	☐	☐	☐	☐	☐
• Andre plager	☐	☐	☐	☐	☐

36. Bruker du medisiner som du har fått av lege på resept? Ja ☐ Nei ☐

37. Bruker du noen av disse medisinene eller kosttilskuddene?
Sett ett kryss for hver linje

	Aldri	Av og til	Nesten daglig
• Jerntabletter	☐	☐	☐
• Avføringstabletter	☐	☐	☐
• Vitaminer	☐	☐	☐
• Tran	☐	☐	☐
• Homeopatmedisin, naturmedisin	☐	☐	☐
• Annet	☐	☐	☐

OM RØYK

38. Røyker noen hjemme hos deg? *Sett ett eller flere kryss*

- Nei, ingen ☐
- Ja, mor ☐
- Ja, far ☐
- Ja, søsken ☐
- Ja, andre ☐

39. Har du prøvd å røyke (minst en sigarett)**?** Ja ☐ Nei ☐

HVIS DU HAR SVART «**NEI**» GÅ TIL SPØRSMÅL **43**

40. **Røyker du selv?**

Sett ett kryss og oppgi eventuelt antall sigaretter.

En pakke tobakk er ca. 50 sigaretter.

- ☐ Ja, jeg røyker ca. ☐☐ sigaretter daglig
- ☐ Ja, jeg røyker av og til, men ikke daglig
- ☐ Nei, ikke nå, men tidligere røykte jeg av og til
- ☐ Nei, ikke nå lenger, men tidligere røykte jeg ca. ☐☐ sigaretter daglig
- ☐ Nei, jeg røyker ikke

HVIS DU HAR SVART **«NEI, JEG RØYKER IKKE»** GÅ TIL SPØRSMÅL **43**

41. **Hvis du røyker eller har røykt daglig:**

- Hvor gammel var du da du begynte å røyke daglig? .... ☐☐ år gammel
- Hvis du har sluttet å røyke daglig, hvor gammel var du da du sluttet? .. ☐☐ år gammel

42. **Hvis du røyker eller har røykt av og til:**

- Hvor gammel var du da du begynte å røyke av og til? .... ☐☐ år gammel
- Hvor mange dager har du røykt den sist måneden? .... ☐☐ ant. dager
  Sett 0 hvis du ikke har røykt den siste måneden
- Omtrent hvor mange sigaretter har du røykt den siste måneden? . ☐☐☐ ant. sigaretter
  Sett 0 hvis du ikke har røykt den siste måneden
- Hvis du har sluttet å røyke av og til, hvor gammel var du da du sluttet? .. ☐☐ år gammel

---

43. **Hvor mange av vennene dine røyker?** Ingen ☐ Noen få ☐ Nesten alle ☐
Sett ett kryss

OM SNUS

44. **Bruker du eller har du brukt snus eller lignende?** *Sett ett kryss*

Nei, aldri ☐ Ja, men jeg har sluttet ☐ Ja, av og til ☐ Ja, hver dag ☐

HVIS DU HAR SVART **«NEI, ALDRI»** GÅ TIL SPØRSMÅL **48**

45. **Hvis du bruker eller har brukt snus:**

- Hvor gammel var du da du begynte med snus? .... ☐☐ år gammel
- Hvis du har sluttet med snus, hvor gammel var du da du sluttet? .... ☐☐ år gammel
- Hvor mange esker snus bruker eller brukte du i måneden? .... ☐☐ antall esker
  Sett 0 hvis du bruker mindre enn en eske i måneden

46. **Hvis du bruker eller har brukt både sigaretter og snus, hva begynte du med først?**
Sett ett kryss

- Snus ☐
- Sigaretter ☐
- Omtrent samtidig (innenfor tre måneder) ☐
- Husker ikke ☐

47. **Begynte du å bruke snus for å prøve å slutte å røyke eller redusere røykingen?**
Sett ett kryss

Nei ☐ Ja, for å slutte å røyke ☐ Ja, for å redusere røykingen ☐

---

48. **Hvor mange av vennene dine bruker snus/skrå?** *Sett ett kryss*

Ingen ☐ Noen få ☐ Nesten alle ☐

49. **Har du noen gang prøvd hasj, marihuana eller andre narkotiske stoffer?**
Sett ett kryss Ja ☐ Nei ☐

Hvis ja, **hvor gammel var du første gang?** ☐ år gammel

50. **Har du venner eller bekjente som bruker narkotiske stoffer?** Ja ☐ Nei ☐

OM IDRETT OG MOSJON

51. Utenom skoletida: **Hvor mange dager i uka driver du idrett, eller mosjonerer du så mye at du blir andpusten og/eller svett?** *Sett bare ett kryss*

- Hver dag ☐
- 4-6 dager i uka ☐
- 2-3 dager i uka ☐
- 1 dag i uka ☐
- Sjeldnere enn en gang i uka ☐
- Sjeldnere enn en gang i måneden ☐
- Aldri ☐

52. Utenom skoletida: **Til sammen hvor mange timer i uka driver du idrett eller mosjonerer du så mye at du blir andpusten og/eller svett?** *Sett bare ett kryss*

- Ingen ☐
- Omtrent ½ time ☐
- Omtrent 1-1½ time ☐
- Omtrent 2-3 timer ☐
- Omtrent 4-6 timer ☐
- 7 timer eller mer ☐

53. **Tenk på de siste 7 dagene: Hvor lang tid brukte du på å sitte en vanlig hverdag?**
Dette kan være tiden du sitter ved PC, gjør lekser, er hos venner, mens du sitter eller ligger og leser eller ser på TV. Regn med tiden du bruker både på skolen og i fritida. ☐ antall timer

54. **Trener du på helsestudio?** Ja ☐ Nei ☐

55. **Hvor ofte har du drevet med følgende treningsaktiviteter de siste 12 månedene?**
Sett ett kryss for hver linje

	Aldri	Under en gang i uka	En gang i uka	Flere ganger i uka
• **Utholdenhetsidrett** (f.eks. løp, langrenn, sykling, svømming)	☐	☐	☐	☐
• **Lag/ballidretter** (f.eks. fotball, vollyball, håndball, ishocky, squash)	☐	☐	☐	☐
• **Estetisk idrett** (f.eks. dans, turn, aerobics)	☐	☐	☐	☐
• **Styrkeidrett** (f.eks. vektløfting, bryting, bodybuilding)	☐	☐	☐	☐
• **Kampsport** (f.eks. judo, karate, taekwondo, boksing)	☐	☐	☐	☐
• **Tekniske idretter** (f.eks. ridning, friidrett, alpint, hopp, snowboard, rullebrett)	☐	☐	☐	☐
• **Risikosport** (f.eks. elvepadling, fjellklatring, paragliding)	☐	☐	☐	☐
• **Lett jogging eller gange/turgåing**	☐	☐	☐	☐
• **Annet**	☐	☐	☐	☐

56. **Hvis du ikke har drevet med noen av disse idrettene de siste 12 månedene, men gjorde det tidligere, hvor gammel var du da du sluttet?**

☐ år gammel

57. **Deltar du i idrettskonkurranser, kamper?** *Sett ett kryss*

Ja ☐ Nei, men jeg deltok før ☐ Nei ☐

ALKOHOL

58. **Har du noen gang prøvd å drikke alkohol?** Ja ☐ Nei ☐ Vet ikke ☐
(Dvs. alkoholholdig øl, vin, rusbrus, brennevin eller hjemmebrent)

Hvis ja, **drikker du av og til alkohol nå?** Ja ☐ Nei ☐

HVIS DU HAR SVART **«NEI»** GÅ TIL SPØRSMÅL **64**

59. **Hvor gammel var du da du begynte å drikke** (mer enn en sup)**?** ☐ år gammel

60. **Har du noen gang drukket så mye alkohol at du har vært beruset (full)?** *Sett ett kryss*

- Nei, aldri ☐
- Ja, en gang ☐
- Ja, 2-3 ganger ☐
- Ja, 4-10 ganger ☐
- Ja,11-25 ganger ☐
- Ja, mer enn 25 ganger ☐

61. **Omtrent hvor mye øl, vin eller brennevin drikker du vanligvis i løpet av to uker?**
Regn ikke med lettøl. Sett 0 hvis du ikke drikker.

- Øl ☐ antall flasker (ca. 3 dl)
- Vin ☐ antall glass (ca. 1 dl)
- Brennevin ☐ antall glass (ca. ½ dl)
- Hjemmebrent ☐ antall glass (ca. ½ dl)
- Rusbrus/cider ☐ antall flasker

92300000019

62. Hvor ofte drikker du alkohol nå for tiden? *Sett ett kryss*

- Hver uke eller oftere ☐
- Hver annen uke ☐
- Sjeldnere enn hver annen uke, men oftere enn en gang i måneden ☐
- En gang i måneden eller sjeldnere ☐
- Aldri ☐

63. På hvilke ukedager drikker du som oftest alkoholholdige drikker? *Sett ett eller flere kryss*

Drikker ikke ☐ Fredager/lørdager ☐ Andre dager i uka ☐

64. Har du noen gang sett at noen av dine foreldre har vært beruset? *Sett ett kryss*

- Aldri ☐
- Noen få ganger ☐
- Noen ganger i året ☐
- Noen ganger i måneden ☐
- Noen ganger i uka ☐

OM KOSTHOLD OG SPISEVANER

65. Hvor ofte spiser du til vanlig disse måltidene? *Sett ett kryss for hver linje*

	Hver dag	4-6 dager i uka	1-3 dager i uka	Sjelden eller aldri
Frokost	☐	☐	☐	☐
Formiddagsmat/nistepakke	☐	☐	☐	☐
Varm middag	☐	☐	☐	☐
Kveldsmat	☐	☐	☐	☐

66. Prøver du å slanke deg? *Sett ett kryss*

Nei, vekten min er passe ☐ Nei, men jeg trenger å slanke meg ☐ Ja ☐

67. Hva pleier du vanligvis å spise på skolen? *Sett ett kryss*

Matpakke ☐ Kjøper mat i kantinen ☐ Spiser ikke på skolen ☐

68. Nedenfor er en liste over ting som gjelder spisevaner. Opplever du noe av dette?
Sett ett kryss for hver linje

	Aldri	Sjelden	Ofte	Alltid
Når jeg først har begynt å spise, kan det være vanskelig å stoppe	☐	☐	☐	☐
Jeg kaster opp etter at jeg har spist	☐	☐	☐	☐
Jeg bruker for mye tid til å tenke på mat	☐	☐	☐	☐
Jeg føler at maten kontrollerer livet mitt	☐	☐	☐	☐
Når jeg spiser, skjærer jeg maten opp i små biter	☐	☐	☐	☐
Jeg bruker lengre tid enn andre på et måltid	☐	☐	☐	☐
Andre mennesker synes at jeg er for tynn	☐	☐	☐	☐
Jeg føler at andre presser meg til å spise	☐	☐	☐	☐

69. **Hvor ofte drikker du vanligvis noe av følgende?** *Sett ett kryss for hver linje*

	Sjelden/ aldri	1-6 glass pr. uke	1 glass pr. dag	2-3 glass pr. dag	4 glass el. mer pr. dag
• Cola/brus/saft med sukker	☐	☐	☐	☐	☐
• Cola/brus/saft uten sukker	☐	☐	☐	☐	☐
• Helmelk/kefir/yoghurt	☐	☐	☐	☐	☐
• Lettmelk/cultura/lettyoghurt	☐	☐	☐	☐	☐
• Skummet melk (sur/søt)	☐	☐	☐	☐	☐
• Fruktjuice	☐	☐	☐	☐	☐
• Vann	☐	☐	☐	☐	☐

70. **Hvor ofte spiser du vanligvis disse matvarene?** *Sett ett kryss for hver linje*

	Flere ganger om dagen	En gang om dagen	Hver uke, men ikke hver dag	Sjeldnere enn hver uke	Aldri
• Grovt brød/knekkebrød	☐	☐	☐	☐	☐
• Feit fisk (laks, ørret, makrell)	☐	☐	☐	☐	☐
• Frukt	☐	☐	☐	☐	☐
• Grønnsaker	☐	☐	☐	☐	☐
• Hvit ost	☐	☐	☐	☐	☐
• Potetgull o.l.	☐	☐	☐	☐	☐
• Sukkertøy, sjokolade, andre søtsaker	☐	☐	☐	☐	☐

71. **Hva slags fett bruker du vanligvis på brødet?** *Sett ett kryss*

Smør/hard margarin ☐ Myk/lett margarin ☐ Flytende margarin/olje ☐ Bruker ikke ☐

72. **Vil du si om deg selv at du er:** *Sett ett kryss*

- Svært tykk .... ☐
- Litt tykk .... ☐
- Omtrent som andre .... ☐
- Heller tynn .... ☐
- Svært tynn .... ☐

HVORDAN HAR DU DET

73. **Når du tenker på hvordan du har det for tida, er du stort sett fornøyd eller er du stort sett misfornøyd?** *Sett ett kryss*

- Svært fornøyd .... ☐
- Meget fornøyd .... ☐
- Ganske fornøyd .... ☐
- Både og .... ☐
- Nokså misfornøyd .... ☐
- Meget misfornøyd .... ☐
- Svært misfornøyd .... ☐

74. **Føler du deg stort sett sterk og opplagt eller trøtt og sliten?** *Sett ett kryss*

- Meget sterk og opplagt .... ☐
- Sterk og opplagt .... ☐
- Ganske sterk og opplagt ...... ☐
- Både og .... ☐
- Ganske trøtt og sliten .... ☐
- Trøtt og sliten .... ☐
- Svært trøtt og sliten .... ☐

92300000019

75. **Er du vanligvis glad eller nedstemt (trist)?** *Sett ett kryss*

- Svært nedstemt (trist) ☐
- Nedstemt (trist) ☐
- Nokså nedstemt (trist) ☐
- Både og ☐
- Nokså glad ☐
- Glad ☐
- Svært glad ☐

76. **Nedenfor er en liste over noen problemer eller plager. Har du vært plaget av noe av dette de siste 14 dagene?** *Sett ett kryss for hver linje*

	Ikke plaget	Litt plaget	Ganske plaget	Veldig plaget
• Vært stadig redd og engstelig	☐	☐	☐	☐
• Følt deg anspent eller urolig	☐	☐	☐	☐
• Følt håpløshet når du tenker på framtida	☐	☐	☐	☐
• Følt deg nedfor eller trist	☐	☐	☐	☐
• Bekymret deg for mye om forskjellige ting	☐	☐	☐	☐

77. **Hva slags oppfatning har du av deg selv?** Kryss av for hver av setningene under ettersom du er enig eller uenig i at de passer for deg. *Sett ett kryss for hver linje*

	Svært enig	Enig	Uenig	Svært uenig
• Jeg har en positiv holdning til meg selv	☐	☐	☐	☐
• Jeg føler meg virkelig ubrukelig til tider	☐	☐	☐	☐
• Jeg føler at jeg ikke har mye å være stolt av	☐	☐	☐	☐
• Jeg føler at jeg er en verdifull person, i hvert fall på lik linje med andre	☐	☐	☐	☐

78. **Hvor ofte opplever du disse reaksjonene som er beskrevet nedenfor?**
Sett ett kryss for hver linje

	Aldri	Sjelden	Noen ganger	Ofte	Alltid
• Jeg føler meg engstelig, og vet ikke hva jeg skal gjøre i en pinlig situasjon	☐	☐	☐	☐	☐
• Jeg føler meg engstelig når jeg er sammen med andre og jeg må gjøre noe mens de ser på meg (f.eks. være med i et skuespill, spille, drive sport)	☐	☐	☐	☐	☐
• Jeg føler meg engstelig når jeg må snakke eller lese høyt foran en gruppe mennesker	☐	☐	☐	☐	☐
• Før jeg skal et sted, der jeg kommer til å være sammen med flere (f.eks. en fest, skolen, fotballkamp) kjenner jeg meg svett, hjertet slår fort og/eller jeg har vondt i hodet eller magen	☐	☐	☐	☐	☐
• Før jeg skal i selskap, eller et sted sammen med andre, tenker jeg på hva som kan gå galt, (f.eks. at jeg gjør feil, virker dum og/eller -tenk hvis de ser hvor redd jeg er!)	☐	☐	☐	☐	☐
• Jeg føler meg engstelig, og vet ikke hva jeg skal gjøre, når jeg er i en ny situasjon	☐	☐	☐	☐	☐

79. **Hvordan har du tenkt og følt om deg selv, og om familien din i løpet av <u>den siste måneden</u>?** *Sett ett kryss for hver linje*

	Helt enig	Litt enig	Middels enig	Litt uenig	Helt uenig
• Jeg får lett andre til å trives sammen med meg ...	☐	☐	☐	☐	☐
• I familien min er vi enige om hva som er viktig i livet ...	☐	☐	☐	☐	☐
• Jeg har lett for å finne nye venner ...	☐	☐	☐	☐	☐
• Jeg trives godt i familien min ...	☐	☐	☐	☐	☐
• Jeg er flink til å snakke med nye folk ...	☐	☐	☐	☐	☐
• Familien min ser positivt på tiden framover selv om det skjer noe veldig leit ...	☐	☐	☐	☐	☐
• Jeg finner alltid noe artig å snakke om ...	☐	☐	☐	☐	☐
• I familien min støtter vi opp om hverandre ...	☐	☐	☐	☐	☐

81. Har du i løpet at den siste måneden:
Sett ett kryss for hver linje

	Nesten hver natt	Ofte	Av og til	Aldri
• Hatt vanskelig for å sovne om kvelden	☐	☐	☐	☐
• Våknet for tidlig og ikke sovnet igjen	☐	☐	☐	☐

82. Har du noen gang opplevd noen av disse hendelsene? *Sett ett kryss for hver linje*

	Nei	Ja, siste året	Ja, i løpet av livet
• At noen i familien din har vært alvorlig syk	☐	☐	☐
• Dødsfall hos noen som sto deg nær	☐	☐	☐
• En katastrofe (brann, ras, flodbølge, orkan e.l)	☐	☐	☐
• En alvorlig ulykke (f.eks. en svært alvorlig bilulykke)	☐	☐	☐
• Blitt utsatt for vold (banket opp eller skadet)	☐	☐	☐
• Sett andre bli utsatt for vold	☐	☐	☐
• Blitt utsatt for ubehagelige seksuelle handlinger av jevnaldrende	☐	☐	☐
• Blitt utsatt for ubehagelige seksuelle handlinger av voksne	☐	☐	☐
• Blitt truet eller fysisk plaget av medelever på skolen i lengere tid	☐	☐	☐
• Fått smertefull eller skremmende behandling på sykehus i forbindelse med sykdom eller skade	☐	☐	☐
• Opplevd noe annet som var veldig skremmende, farlig eller voldelig	☐	☐	☐

HVIS DU HAR SVART «**NEI**» PÅ ALLE HENDELSENE GÅ TIL SPØRSMÅL **87**

Dersom du har opplevd noen slik hendelse:

83. Tenker du fortsatt mye på det som skjedde? Ja ☐ Nei ☐

Hvis ja, **har du skremmende tanker, ser for deg bilder eller hører lyder fra det som skjedde, selv om du ikke vil det?** Ja ☐ Nei ☐

84. Når noe minner deg om det som skjedde, blir du veldig ute av deg, redd eller trist? Ja ☐ Nei ☐

85. Forsøker du å ikke snakke om det, tenke på det eller ha følelser rundt det som skjedde? Ja ☐ Nei ☐

86. Hvis du har vært utsatt for en skade eller ulykke, har du fått fysiske (kroppslige) senfølger eller plager etterpå? Ja ☐ Nei ☐

OM FRITIDA

87. **Hvor mange lag eller foreninger er du med i** (f.eks. idrettslag, speiderforening, musikk-korps)**?**

Ingen ☐ En ☐ To eller flere ☐

88. **Hvor ofte gjorde du noen av disse aktivitetene i løpet av den siste uka?**
Sett ett kryss for hver linje

	Ingen	1 gang	2-3 ganger	4 ganger eller flere
• Besøkte noen du kjente	☐	☐	☐	☐
• Fikk besøk hjemme	☐	☐	☐	☐
• Leste en bok, blad, tegneserie	☐	☐	☐	☐
• Hørte på musikk	☐	☐	☐	☐
• Spilte et instrument	☐	☐	☐	☐
• Var ute mer enn 2 timer av gangen med venner	☐	☐	☐	☐
• Var på møte eller trening i en forening eller et lag	☐	☐	☐	☐
• Drev med en annen hobby	☐	☐	☐	☐
• Gjorde lekser eller hjemmearbeid lenger enn 1 time	☐	☐	☐	☐
• Så på TV/DVD	☐	☐	☐	☐
• Spilte PC/TV spill	☐	☐	☐	☐
• Spilte, chattet eller surfet på internett	☐	☐	☐	☐
• Var på biblioteket	☐	☐	☐	☐
• Var på kino	☐	☐	☐	☐
• Var på kafe eller annet møtested for ungdom	☐	☐	☐	☐
• Spilte teater/revy	☐	☐	☐	☐
• Drev med film/foto	☐	☐	☐	☐
• Var på konsert	☐	☐	☐	☐
• Var tilskuer på idrettsarrangement	☐	☐	☐	☐
• Sang i kor	☐	☐	☐	☐

89. **Dersom du vanligvis driver med noen av disse aktivitetene: hvor lenge pleier du å holde på hver gang?** *Sett ett kryss for hver linje*

	Mindre enn ½ time	½ -1 time	Mer enn 1 time
• Ser på TV/DVD	☐	☐	☐
• Spiller PC/TV spill	☐	☐	☐
• Spiller, chatter eller surfer på nettet	☐	☐	☐
• Hører på musikk	☐	☐	☐

90. **Har du mobiltelefon?** Ja ☐ Nei ☐

Hvis ja:

• Hvor lenge snakker du vanligvis i mobiltelefonen i løpet av dagen? . ☐ ant. minutter

• Hvor mange meldinger får du vanligvis i døgnet? .... ☐ ant. meldinger

• Hvor mange meldinger sender du vanligvis i døgnet? .... ☐ ant. meldinger

OM FAMILIE OG VENNER

91. **Omtrent hvor mange nære venner har du?** (Regn med de du kan snakke fortrolig med og som kan gi deg god hjelp når du trenger det. Regn ikke med de du bor sammen med, men regn med andre slektninger.) *Sett ett kryss*

Ingen ☐ En ☐ To eller flere ☐

92. **Har du fast kjæreste?** Ja ☐ Nei, ikke nå, men før ☐ Nei ☐

93. **Er foreldrene dine separert eller skilt, eller har de noen gang vært fra hverandre for mer enn ett år?** *Sett ett kryss og eventuelt alderen din*

☐ Nei

☐ Ja, de flyttet fra hverandre eller ble separert da jeg var .... ☐☐ år gammel, men flyttet senere sammen igjen

☐ Ja, de ble skilt eller flyttet fra hverandre for godt da jeg var .... ☐☐ år gammel.

94. **Hvor god råd synes du familien din har i forhold til de fleste andre?** *Sett ett kryss*

Omtrent som de fleste andre ☐ Bedre råd ☐ Dårligere råd ☐

95. **Har det vært eller er det ofte krangling i familien din?** *Sett ett kryss*

Nei ☐ Ja, siste 12 mnd ☐ Ja, tidligere ☐

96. **Hvor godt forhold føler du at du har til den nære familien din?** Sett et kryss for de familiemedlemmene som er aktuelle for deg. Hvis du har flere søsken, tenk på den du har det beste forholdet til. *Sett ett kryss for hver linje*

	Svært godt	Godt	Ikke så godt	Dårlig
• Mor	☐	☐	☐	☐
• Far	☐	☐	☐	☐
• Søsken	☐	☐	☐	☐
• Stemor eller stefar	☐	☐	☐	☐

97. **Hender det ofte at du føler deg ensom?** *Sett ett kryss*

- Svært ofte .... ☐
- Ofte .... ☐
- Av og til .... ☐
- Sjelden .... ☐
- Svært sjelden eller aldri .... ☐

OM SKOLEN

98. Hender noe av dette deg på skolen nå, eller har det hendt før? *Sett ett kryss for hver linje*

	Aldri	En gang i blant	Ofte	Svært ofte
• Har vanskelig for å konsentrere deg i timen	☐	☐	☐	☐
• Synes gym eller formingstimene er morsomme	☐	☐	☐	☐
• Synes andre timer er morsomme	☐	☐	☐	☐
• Krangler med læreren	☐	☐	☐	☐
• Gleder deg til å gå på skolen	☐	☐	☐	☐
• Skulker	☐	☐	☐	☐
• Forstår når lærerne underviser	☐	☐	☐	☐
• Har det morsomt i friminuttene	☐	☐	☐	☐
• Er fornøyd med resultatene på prøver	☐	☐	☐	☐
• Kommer i slåsskamp	☐	☐	☐	☐
• Får skjenn av læreren	☐	☐	☐	☐
• Klarer ikke å være rolig i timene	☐	☐	☐	☐
• Kjeder deg, eller mistrives	☐	☐	☐	☐
• Får hjelp med lese og skrivevansker	☐	☐	☐	☐
• Blir kalt noe negativt av andre elever	☐	☐	☐	☐
• Blir holdt utenfor av andre elever	☐	☐	☐	☐

OM HELSETJENESTEN

99. Har du i løpet av de siste 12 månedene vært hos: *Sett ett kryss for hver linje*

	Ja	Nei
• Allmennpraktiserende lege (fastlegen, lege utenom sykehus)	☐	☐
• Lege på sykehus	☐	☐
• Helsestasjon for ungdom	☐	☐
• Skolehelsetjenesten	☐	☐
• Psykolog	☐	☐
• Fysioterapeut	☐	☐
• Kiropraktor	☐	☐
• Annen behandler (homøopat, naturmedisiner, fotsoneterapeut, håndspålegger, "healer", "synsk" e.l.)	☐	☐

100. Har du vært innlagt på sykehus i løpet av de siste 12 månedene?

Ja ☐ Nei ☐

101. Hvor ofte har du vært borte fra skolen p.g.a. sykdom de siste 12 månedene?

Mindre enn en uke ☐ 1-2 uker ☐ Mer enn 2 uker ☐

OM UTVIKLING

Her er noen spørsmål om kroppslige forandringer som skjer gjennom ungdomstiden:

102. Når man er tenåring, er det perioder da man vokser raskt. Har du merket at kroppen din har vokst raskt (blitt høyere)**?** *Sett ett kryss*

- Nei, den har ikke begynt å vokse raskt .... ☐
- Ja, den har så vidt begynt å vokse raskt .... ☐
- Ja, den har helt tydelig begynt å vokse raskt .... ☐
- Ja, det virker som om jeg er ferdig med å vokse raskt .... ☐

103. Og hva med hår på kroppen (under armene og i skrittet)**? Vil du si at håret på kroppen din har:** *Sett ett kryss*

- Ikke begynt å vokse enda .... ☐
- Såvidt begynt å vokse .... ☐
- Helt tydelig begynt å vokse .... ☐
- Det virker som om håret på kroppen er utvokst .... ☐

104. Når du ser på deg selv nå, mener du at du er/var tidligere eller senere fysisk moden enn andre på din alder? *Sett ett kryss*

- Mye tidligere .... ☐
- Noe tidligere .... ☐
- Lite grann tidligere .... ☐
- Akkurat som andre .... ☐
- Lite grann senere .... ☐
- Noe senere .... ☐
- Mye senere .... ☐

SPØRSMÅL FOR GUTTER

105. Har du begynt å komme i stemmeskiftet? *Sett ett kryss*

- Nei, har ikke begynt ennå .... ☐
- Ja, har så vidt begynt .... ☐
- Ja, har helt tydelig begynt .... ☐
- Det virker som om stemmeskiftet er ferdig .... ☐

106. Har du begynt å få bart eller skjegg? *Sett ett kryss*

- Nei, har ikke begynt ennå .... ☐
- Ja, har så vidt begynt .... ☐
- Ja, har helt tydelig begynt .... ☐
- Ja, har fått en god del skjeggvekst .... ☐

SPØRSMÅL FOR JENTER

107. Har du begynt å få bryster? *Sett ett kryss*

- Nei, har ikke begynt ennå ...... ☐
- Ja, har så vidt begynt .... ☐
- Ja, har helt tydelig begynt .... ☐
- Det virker som om brystene er fullt utviklet ☐

108. Har du fått menstruasjon («mensen»)**?** Ja ☐ Nei ☐

HVIS DU HAR SVART **«NEI»** GÅ TIL SPØRSMÅL **113**

109. Hvor gammel var du da du fikk din første menstruasjon? Jeg var ☐ år og ☐ måneder

110. Hvor mange menstruasjoner har du hatt de siste 12 månedene? ☐ antall

111. Hvor lenge er det vanligvis mellom menstruasjonene dine?
(Fra første dag i en menstruasjon til første dag i neste)

Mindre enn 3 uker ☐ 3-4 uker ☐ Mer enn 4 uker ☐

112. Har du noen gang etter en menstruasjonsblødning vært blødningsfri i flere måneder?
(Uten å ha vært gravid) *Sett ett kryss*

- Ja, 2-5 mnd .... ☐
- Ja, 6-12 mnd .... ☐
- Ja, mer enn 1 år .... ☐
- Nei, aldri .... ☐

---

113. Har du noen gang brukt p-piller eller minipiller?

Ja, jeg bruker det nå ☐ Ja, jeg har brukt det før ☐ Nei ☐

Hvis ja:

Hvor gammel var du første gang du brukte p-piller/minipiller? ..... ☐ år gammel

Hvor mange år har du brukt p-piller/minipiller til sammen? .... ☐ år til sammen
Sett 0 hvis du har brukt p-piller/minipiller mindre enn ett år

FOR ELEVER I VIDEREGÅENDE SKOLE

Disse spørsmålene skal bare besvares av de som går i videregående skole:

114. **Har du i løpet av det siste året ofte følt at du har presset deg, eller stadig drevet deg selv framover?**

Ja ☐ Nei ☐ Vet ikke ☐

115. **Føler du deg under tidspress, også når det gjelder daglige gjøremål?**

Alltid, eller nesten alltid ☐ Noen ganger ☐ Aldri ☐

116. **Har du hatt tanker om å ta ditt eget liv?** Ja ☐ Nei ☐

117. **Har du noen gang brukt anabole steroider eller andre dopingmidler?**

Ja ☐ Nei ☐

---

118. **Har du noen gang hatt samleie?** Ja ☐ Nei ☐

Hvis ja, **hvor gammel var du første gang?** .... ☐ år gammel

119. For JENTER: **Har du noen gang vært gravid uten at du ønsket det?**

Ja ☐ Nei ☐

120. For GUTTER: **Har en jente noen gang blitt gravid med deg uten at det var meningen?**

Ja ☐ Nei ☐ Vet ikke ☐

Hvis ja: (for både gutter og jenter)

Hvor gammel var du da dette skjedde? .... ☐ år gammel

Ble det utført abort? Ja ☐ Nei ☐ Vet ikke ☐

KOMMENTARER

Hvis du har tid, kan du gjerne skrive litt om det du synes er viktig, men som det ikke er spurt etter i spørreskjemaet. Hvordan synes du det er å være ung i dag? Er det noe du mener kan bli bedre når det gjelder helse og trivsel for dere som er unge?

Tusen takk for innsatsen!

92300000019

Foto: Harald Sæterøy og Johan Arnt Nesgård

HUNT forskningssenter

HUNT forskningssenter ligger i Verdal og er en del av Det medisinske fakultet, NTNU.
HUNT forskningssenter gjennomfører befolkningsundersøkelser i Nord-Trøndelag, forvalter forskningsdata og driver medisinsk og helsefaglig forskning.

Hvis du har spørsmål om Ung-HUNT 3 kan du ta kontakt med:
HUNT forskningssenter • Neptunveien 1, 7650 Verdal
Telefon: 74 07 51 80 • Faks: 75 07 51 81 • hunt@medisin.ntnu.no • www.hunt.ntnu.no

hunt 3
Helseundersøkelsen i Nord-Trøndelag

NTNU
Det skapende universitet
