# Supplementary material for: Risk factors and risk profiles for neck pain in young adults: Prospective analyses from adolescence to young adulthood—The North-Trøndelag Health Study
Source: PLoS One. 2021 Aug 12;16(8):e0256006. doi: 10.1371/journal.pone.0256006 (PMC8360564; doi:10.1371/journal.pone.0256006)
Supplement: S4 Questionnaire — (PDF) [file pone.0256006.s007.pdf]

Young HUNT

ADOLESCENT SECTION OF THE HEALTH STUDY IN NORD-TRØNDELAG, HUNT

It's your turn to participate in the Nord-Trøndelag Health Study (**HUNT**)!

We hope you have read the information brochure about YOUNG HUNT that you took home with you and have decided to participate!

Read the informed consent form that is inside the questionnaire and check that it is your name that is on it. Mark it as to whether you will participate or not, sign it and hand it in to the teacher.

Your name should NOT be on your questionnaire!

Put an X in the boxes ∀ that you think apply to you. Answer the best you can! If there are questions that you do not want to answer, skip them.

When you are finished, put the questionnaire in the envelope you have been given, seal it and give the envelope to the teacher. Do this even if you haven't finished the questionnaire.

All your answers will be treated in the strictest of confidence!

No one at school is allowed to see your answers.

If you wish to speak to someone about the study, speak to the Young HUNT nurse when she visits your school or ring HUNT Research Centre (see back of questionnaire).

Good Luck and Thank You!

Date of questionnaire completion ____/_____20____

1. For those who are in Junior High School: **What type of plans do you have regarding your studies in High School?**

High School academic studies ∀ High School vocational studies ∀ Don't know ∀

2. What type of plans do you have regarding continued studies?
(Put one or more Xs)

* College or university for 4 years or more ………………… ∀
* College or university less than 4 years ………………. ∀
* Other vocational training …………∀
* No plans …………………………..…∀
* Don't know…………………………∀

WHERE YOU LIVE

3. What type of housing do you live in? (Only one X)

* Single-family house ……………..…∀
* Row house/2-4 family housing ……∀
* Flat in block/flat …………………….∀
* Farm w/ animal husbandry.……………∀
* Farm w/out animal husbandry …………∀
* Other housing ……………………∀

4. Who do you currently live with? (Put one or more Xs)

* Mother ……………………………∀
* Father ……………………………..∀
* 1-2 siblings ………………….… ….∀
* 3 or more siblings………………… ∀
* Mother's new husband or partner ∀
* Father's new wife or partner………∀
* Foster parents…………………∀
* Adoptive parents ……………….∀
* Grandparents/other…………….∀
* Spouse/partner…………………∀
* Friends…………………………..∀
* Alone/in a rented room……….. ∀

5. If your mother and father do not live together, who do you live with?

Mostly my mother ∀ Mostly my father ∀ Equal time at both parents ∀

6. Are there pets living in your home?

No …………………… ∀
Yes, cat ……………… ∀
Yes, dog …………….. ∀
Yes, other animals with fur ……… ∀
Yes, bird …………………………… ∀
Yes, other …………………………… ∀

YOUR HEALTH

7. How is your health at the moment? (One X)

* Poor ……………………………………∀
* Not so good ……………………………∀
*Good……………………..∀
* Very good………………∀

8. Are you disabled in any of these ways? (Put an X for each line)

	No	A little	Somewhat	Severely
* Motor impairment (movement)	∀	∀	∀	∀
* Vision impairment	∀	∀	∀	∀
* Hearing impairment	∀	∀	∀	∀
* Impairment due to physical illness	∀	∀	∀	∀
* Impairment due to mental health problems	∀	∀	∀	∀

9. Have you had any of these ailments <u>in the past 12 months</u>: (Put an X for each line)

	Not at all	A little	Much
* Palpitation	∀	∀	∀
* Constipation	∀	∀	∀
* Diarrhoea	∀	∀	∀
* Alternating constipation and diarrhoea	∀	∀	∀
* Bloating	∀	∀	∀
* Nausea	∀	∀	∀

ALLERGIES

10. Do you have allergies? Yes ∀ No ∀ Don't know ∀

If Yes, **what do you think you are allergic to?** (One or more Xs)

* Grass/trees ∀
* House dust ∀
* Dogs ∀
* Cats ∀
* Horses ∀
* Food ∀
* Other ∀
* Don't know ∀

11. Has a doctor given you any allergy tests (blood tests, skin tests)**?**
Yes ∀ No ∀ Don't know ∀

If Yes, **what did you have an allergic reaction to?** (One or more Xs)

* Nothing	∀	* Dog	∀	* Food	∀
* Grass/trees	∀	* Cat	∀	* Other	∀
* House dust	∀	* Horse	∀	* Don’t know	∀

RESPIRATORY TRACT

12. Have you ever had wheezing or whistling in the chest?

Yes ∀ No ∀

IF YOU ANSWERED “NO”, SKIP TO QUESTION 15

13. Have you had wheezing or whistling in the chest <u>in the past 12 months</u>?

Yes ∀ No ∀

IF YOU ANSWERED “NO”, SKIP TO QUESTION 15

14. How many attacks of wheezing have you had <u>in the past 12 months</u>?

None ∀ 1 to 3 ∀ 4 to 12 ∀ More than 12 ∀

****

15. Do you have or have you had asthma? Yes ∀ No ∀

If YES, **has a doctor said that you have/have had asthma?** Yes ∀ No ∀

16. In the past <u>12 months</u> has your chest sounded wheezy during or after exercise?

Yes ∀ No ∀

17. <u>In the last 12 months</u> have you had a dry cough at night apart from a cough associated a cold or chest infection?

Yes ∀ No ∀

NASAL PROBLEMS

18. <u>In the past 12 months</u>, have you had a problem with sneezing or a runny or blocked nose when you did not have a cold or the flu?

Yes ∀ No ∀

IF YOU ANSWERED “NO”, SKIP TO QUESTION 21

19. Has this nose problem been accompanied by itchy-watery eyes?

Yes ∀ No ∀

20. How much did this nose problem interfere with your daily activities? (One X)

Not at all ∀ A little∀ A moderate amount ∀ A lot ∀

21. Have you ever had hay fever or nasal allergies? Yes ∀ No ∀

RASHES

22. Have you had an itchy rash during <u>the last 12 months</u>? Yes ∀ No ∀

IF YOU ANSWERED "NO", SKIP TO QUESTION 25

23. Have you had this itchy rash in the following places: the folds of your elbow (inside), back of your knees, on the front of your ankles, under your buttocks or around your neck, ears or eyes? Yes ∀ No ∀

24. How often on the average has this itchy rash kept you awake at night? (One X)

Not at all ∀ Less often than 1 night a week ∀ 1 night or more a week ∀

---

25. Have you ever had eczema? Yes ∀ No ∀

If Yes, has a doctor said that you have/ have had "atopic eczema"? Yes ∀ No ∀

ACNE

26. Have you had problems with acne? Yes ∀ No ∀

IF YOU ANSWERED "NO", SKIP TO QUESTION **31**

27. Where was the acne? (Put one or more Xs)

Forehead…**…..**∀ Cheeks**……..**∀ Shoulders**……..**∀ Other places**……...**∀

Nose**…………**∀ Chest**………..**∀ Back**……………**∀

28. How much has the acne bothered you? Very much ∀ Much ∀ A little ∀ Not at all ∀

Only one X

29. Have you used non-prescription creams, skin astringents or other similar products to get rid of the acne? (bought at the drug store or other shop, not prescribed by a doctor)

Yes ∀ No ∀

If Yes, has it helped? One X No ∀ Some ∀ Yes ∀

30. Have you been to a doctor because of acne? Yes ∀ No ∀

If Yes, did the doctor recommend any of the following treatments? (Put an X for each line)

- Topical treatment (ex: creams or liquid solutions) …………… Yes ∀ No ∀
- Antibiotic tablets (tetracycline) ………………………………….. Yes ∀ No ∀
- Roaccutan tablets ………………………………………………… Yes ∀ No ∀

If Yes, did this treatment help? (One X) No ∀ Some ∀ Yes ∀

PAIN

31. How often have you had any of the below listed pain <u>during the last 3 months</u>? (Without having injured yourself or having a known illness that is the reason for the pain)
Look at the figure and put an X for each line

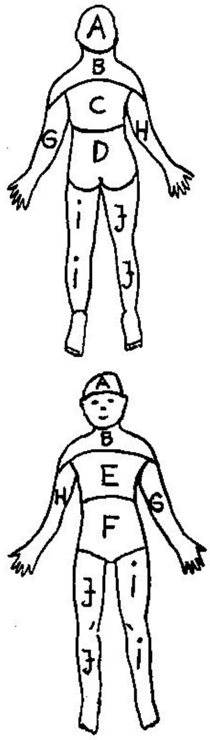

	Never or seldom	About once a month	About once a week	More than once a week	Almost every day
A. Headache/migraine					
B. Neck/ shoulder pain					
C. Pain in the upper back					
D. Pain in the lower back/buttocks					
E. Pain in chest					
F. Stomach pain					
G. Pain in left arm					
H. Pain in right arm					
I. Pain in left leg					
J. Pain in right leg					
Other pain					

IF YOU ANSWERED “NEVER OR SELDOM” FOR EVERYTHING, SKIP TO QUESTION 34

If you have had pain during the last 3 months,

32. Does anything on the below list apply to you? (Put an X for each line):

	Yes	No
* Pain makes it difficult to fall asleep……………………...…………………………..	∀	∀
* Pain disturbs my sleep at night. ……………………………………………………….	∀	∀
* Pain makes it difficult to sit in class. ……………………… ………………………..	∀	∀
* Pain makes it difficult for me to walk more than one kilometre. ……… ………….	∀	∀
* Because of pain I have problems in gym class. ……………………………………..	∀	∀

33. All things considered, has pain made it difficult to do daily activities? (Put an X for each line)

	No	Yes, sometimes	Yes, often
* At school ………………………………………………………………	∀	∀	∀
* In leisure time ………………………………………………………	∀	∀	∀

If you answered Yes, **what type of pain makes daily activities difficult?** *(One or more Xs)*

Headache/migraine ∀ Stomach pain ∀ Muscular/skeletal pain ∀ Other pain ∀

OTHER ILLNESSES

34. Has a doctor diagnosed you with: (Put an X for each line)

	Yes	No
* Epilepsy	∀	∀
* Diabetes	∀	∀
* Migraine	∀	∀
* Juvenile arthritis	∀	∀
* Other illnesses that have lasted longer than 3 months	∀	∀

MEDICINE USE

35. How often <u>in the last 3 months</u> have you taken non-prescription medicine for any of the below listed complaints? (medicine not prescribed by a doctor, for example bought at a store or pharmacy) (Put an X for each line)

	Never	1 day a week or less	2 days a week	3 days a week	4 days a week or more
* Headache/migraine	∀	∀ ∀	∀	∀	
* Muscle/joint pain	∀	∀ ∀	∀	∀	
* Back pain	∀	∀ ∀	∀	∀	
* Stomach pain	∀	∀ ∀	∀	∀	
* Other	∀	∀ ∀	∀	∀	

36. Do you take any medicine that was prescribed for you by a doctor? Yes ∀ No ∀

37. Do you take/use any of these medicines or dietary supplements?
(Put an X for each line)

	Never	Sometimes	Almost daily
* Iron tablets	∀	∀	∀
* Laxative tablets	∀	∀	∀
* Vitamins	∀	∀	∀
* Cod-liver oil	∀	∀	∀
* Homeopathic medicine, herbal medicine	∀	∀	∀
* Other	∀	∀	∀

TOBACCO

38. Does anyone you live with smoke at home? (One or more Xs)

* No, nobody ∀
* Yes, my mother ∀
* Yes, my father ∀
* Yes, a sibling ∀
* Yes, other people ∀

39. Have you tried smoking? (at least one cigarette) Yes ∀ No ∀

IF YOU ANSWERED "NO", SKIP TO QUESTION 43

40. Do you smoke? (Put an X in the appropriate box and write in the number of cigarettes. A package of loose tobacco equals approx. 50 cigarettes)

∀ Yes, I smoke about ____________ cigarettes daily.

∀ Yes, I smoke occasionally, but not daily.

∀ No, not anymore, but previously I smoked occasionally.

∀ No, not anymore, but previously I smoked about ________cigarettes daily.

∀No, I don't smoke.

IF YOU ANSWERED "NO, I DON'T SMOKE", SKIP TO QUESTION 44

41. If you smoke or have smoked daily:

* How old were you when you began smoking daily? ______ years old

* If you quit smoking daily, how old were you when you quit? ______ years old

42. If you smoke or have smoked occasionally:

* How old were you when you began smoking occasionally? ______ years old

* How many days have you smoked in the last month? ______ number of days
(Write 0 if you have not smoked in the past month)

* About how many cigarettes have you smoked in the last month? ______ number of cigarettes
(Write 0 if you have not smoked in the past month)

* If you quit smoking occasionally, how old were you when you quit? ______ years old

43. How many of your friends smoke? None ∀ A few ∀ Almost all ∀
(One X)

****

44. Do you use or have you used snuff, chewing tobacco or similar products? (One X)

No, never ∀ Yes, but have quit ∀ Yes, sometimes ∀ Yes, everyday ∀

IF YOU ANSWERED "NO, NEVER", SKIP TO QUESTION 50

45. If you use or have used snuff/chewing tobacco:

* How old were you when you began using snuff/chewing tobacco? _____ years old

* If you stopped using snuff/chewing tobacco, how old were you when you stopped? _____years old

* How many boxes/bags of snuff/chewing tobacco do you use/have you used a week?
_____ number of boxes/bags
(Write 0 if you use less than one box a month

46. If you smoke cigarettes and use snuff, which did you start first?
(One X)

∀ Snuff ∀ About the same time (within 3 months)
∀ Cigarettes ∀ Don't remember

47. Did you start using snuff to try to quit smoking or to smoke less?
(One X)

∀ No ∀ Yes, to quit smoking ∀ Yes, to smoke less

48. How many of your friends use snuff/chewing tobacco? (One X**)**
None ∀ A few ∀ Almost all ∀

****

49. Have you ever tried hash, marijuana or other drugs? (One X) Yes ∀ No ∀

If Yes, **How old were you the first time?** _______ years old

50. Do you have friends or acquaintances who use drugs? Yes ∀ No ∀

SPORTS AND EXERCISE

51. Not during the average school day: **How many <u>days</u> a week do you play sports or exercise to the point where you breathe heavily and/or sweat?** (*Only one X*)

* Everyday ∀
* 4-6 days a week ∀
* 2-3 days a week ∀
* 1 day a week ∀
* Less often than once a week ∀
* Less often than once a month ∀
* Never ∀

52. Not during the average school day: **How many __hours__ a week do you play sports or exercise to the point where you breathe heavily and/or sweat?** (*Only one X*)

None	∀	* About 2-3 hours	∀
About ½ hour	∀	* About 4-6 hours	∀
About 1-1½ hours	∀	* 7 or more hours	∀

53. Think about __the past 7 days__: How many hours did you spend sitting in an average day? (This could be the time spent sitting at the computer, doing homework, at friends, reading and TV watching (include times both sitting and laying down for the last two). Count the times at school and in your leisure time.) ___________Number of hours

54. Do you work out/train at a health club? Yes ∀ No ∀

55. How often have you done/participated in any of the following activities/sports __the past 12 months__? (Put an X for each line)

	Never	Less than 1 x a week	Once a week	Several x a week
* Endurance sports (ex: running, cross-country skiing, cycling, swimming)	∀	∀	∀	∀
* Team sports (ex: football, volleyball, handball, ice hockey, squash)	∀	∀	∀	∀
* Aesthetic sports (ex: dance, gymnastics, aerobics)	∀	∀	∀	∀
* Strength sports (ex: weightlifting, wrestling, bodybuilding)	∀	∀	∀	∀
* Martial arts/combat sports (ex: judo, karate, taekwondo, boxing)	∀	∀	∀	∀
* Technical sports (ex: riding, track sports, alpine skiing, ski jumping, snowboard, skate boarding)	∀	∀	∀	∀
* Adrenaline sports (ex: white water rafting, mountain climbing, paragliding)	∀	∀	∀	∀
* Jogging or racewalking/hiking	∀	∀	∀	∀
* Other	∀	∀	∀	∀

56. If you haven't been involved in any of these activities/sports in the past 12 months, but did so previously, how old were you when you stopped? _____ years old

57. Do you participate in sports competitions? (One X)

Yes ∀ No, but I used to compete ∀ No ∀

ALCOHOL

58. Have you ever tried drinking alcohol? (Meaning alcoholic beer, wine, hard liquor or moonshine)

Yes ∀ No ∀ Don't know ∀

If Yes, **do you sometimes drink alcohol now?** Yes ∀ No ∀

IF YOU ANSWERED NO, SKIP TO QUESTION 66

59. **How old were you when you began drinking** (more than a sip)**?** ________ years old

60. **Have you ever drunk so much alcohol that you felt intoxicated (drunk)?**
(One X)

* No, never ……………. ∀
* Yes, once …………… ∀
* Yes, 2-3 times……… ∀
* Yes, 4-10 times ……………………….. ∀
* Yes, 11-25 times …….. ………………. ∀
* Yes, more than 25 times .... ∀

61. **About how much beer, wine or hard liquor do you usually drink during <u>two weeks</u>?** Don't count alcohol free beer. Write 0 if you do not drink alcohol.

Beer........... number of 1/2 bottles

Wine.......... number of glasses (approx. 1 dl)

Hard liquor, liqueurs........... number of glasses (approx. 1/2 dl)

Moonshine .... number of glasses (approx. 1/2 dl)

Alcopop ………………. number of bottles

62. **How often do you currently drink alcohol?** (One X)

* Every week or more often ……………………………………………………………………. ∀
* Every other week …………………………………………………………………………….. ∀
* More seldom than every other week, but more often than once a month ………………… ∀
* Once a month or more seldom than once a month ……..…………………………….... ∀
* Never …………………………………………………………………………………….. ∀

63. **On which days during the week do you most often drink alcohol?** (One or more Xs)

I do not drink ∀ Fridays/Saturdays ∀ Other days of the week ∀

64. **Have you ever seen either of your parents intoxicated?** (One X)

* Never ……………..∀
* A few times ………∀
* A few times during the year ………∀
* A few times a month ………………∀
* A few times a week ……………….∀

MEALS AND EATING HABITS

65. How often do you usually eat these meals? *(Put an X for each line)*

	Every-day	4-6 days a week	1-3 days a week	Seldom or never
* Breakfast	∀	∀	∀	∀
* Lunch	∀	∀	∀	∀
* Dinner (warm)	∀	∀	∀	∀
***** Supper/evening snack	∀	∀	∀	∀

66. Are you trying to lose weight? (*One X)*

No, I'm comfortable with my weight ∀ No, but I need to lose weight ∀ Yes ∀

67. What do you usually eat at school? (*One X)*

Packed lunch ∀ Buy food at the cafeteria ∀ Do not eat lunch at school ∀

68. Below are listed things that concern your eating habits. (Put an X for each line*)*

	Never	Seldom	Often	Always
* When I first begin eating, it is difficult to stop.	∀	∀	∀	∀
* I vomit after I have eaten.	∀	∀	∀	∀
* I spend too much time thinking about food.	∀	∀	∀	∀
* I feel that food controls my life.	∀	∀	∀	∀
* When I eat, I cut my food up in small pieces.	∀	∀	∀	∀
* It takes me longer than others to finish a meal.	∀	∀	∀	∀
* Other people think I'm too thin.	∀	∀	∀	∀
* I feel that others pressure me to eat.	∀	∀	∀	∀

69. How often do you usually drink the following? (Put an X for each line)

	Seldom/ never	1-6 glasses a week	1 glass a day	2-3 glasses a day	4 glass or more a day
* Cola/soda/still soft drinks w/ sugar……	∀	∀	∀	∀	∀
* Cola/soda/still soft drinks w/out sugar…	∀	∀	∀	∀	∀
* Whole milk/kefir/yoghurt………………….	∀	∀	∀	∀	∀
* Low fat milk or yoghurt/cultured milk…….	∀	∀	∀	∀	∀
* Skim milk (sour/sweet) ...……………….	∀	∀	∀	∀	∀
* Fruit juice ……………………………….	∀	∀	∀	∀	∀
* Water …… …………………………….	∀	∀	∀	∀	∀

70. How often do you usually eat the following foods? (Put an X for each line)

	Several times a day	Once a day	Every week but not everyday	Less often than every week	Never
* Whole grain bread/crispbread ……………	∀	∀	∀	∀	∀
* Oily fish (salmon, trout, mackerel)………	∀	∀	∀	∀	∀
* Fruit………………………………………..	∀	∀	∀	∀	∀
* Vegetables ……………………………….	∀	∀	∀	∀	∀
* White cheese ……………………………….	∀	∀	∀	∀	∀
* Potato chips and such …………………….	∀	∀	∀	∀	∀
* Candy, chocolate, other sweets……..…..	∀	∀	∀	∀	∀

71. What type of fat do you usually use on bread? (One X)

Butter/hard margarine ∀ Soft/low fat margarine ∀ Liquid margarine/Oil ∀ Don't use any ∀

72. Do you consider yourself: (One X)

* Very fat …………………… ∀
* Chubby ………………………. ∀
* About the same as others…… ∀
* Thin…………………… ∀
* Very thin…………….. ∀

HOW THINGS ARE GOING FOR YOU

73. Thinking about your life at the moment, would you say that you by and large are satisfied with life, or are you mostly dissatisfied? (One X)

* Very satisfied ……………….. ∀
* Satisfied…….. ……………….. ∀
* Somewhat satisfied ………….. ∀
* Neither satisfied nor dissatisfied ∀
* Somewhat dissatisfied ……… ∀
* Dissatisfied ………………….. ∀
* Very dissatisfied …………….. ∀

74. Do you feel, for the most part, strong and fit or tired and worn out? (One X)

* Very strong and fit …………….∀
* Strong and fit …………………..∀
* Somewhat strong and fit ……..∀
* Somewhere in between ………∀
* Somewhat tired and worn out..∀
* Tired and worn out…………….∀
* Very tired and worn out ….∀

75. Would you say you are usually cheerful or downhearted (sad)? (One X)

* Very downhearted (sad) ………………. ∀
* Downhearted (sad) ……………………. ∀
* Somewhat downhearted (sad) ………. ∀
* Some of both …………………………... ∀
* Somewhat cheerful ………….. ∀
* Cheerful ………………………. ∀
* Very cheerful …………………. ∀

76. Below is a list of some problems. Have you been bothered by any of these in <u>the last 14 days</u>? (Put an X for each line)

	Not bothered	A little bothered	Quite bothered	Very bothered
* Been constantly afraid and anxious ……………………	∀	∀	∀	∀
* Felt tense or uneasy ……………………………………	∀	∀	∀	∀
* Felt hopelessness when you think of the future ………..	∀	∀	∀	∀
* Felt dejected or sad …………………………………….	∀	∀	∀	∀
* Worried too much about various things …………………	∀	∀	∀	∀

77. How do you see yourself? Put an X in a box for each sentence below indicating whether you agree or disagree in how it relates to you. (Put an X for each line)

	Strongly Agree	Agree	Disagree	Strongly disagree
* I take a positive attitude toward myself…………………….	∀	∀	∀	∀
* I certainly feel useless at times……………………………..	∀	∀	∀	∀
* I feel I do not have much to be proud of…………………..	∀	∀	∀	∀
* I feel that I am a person of worth, at least on an equal plane with others…..…………………	∀	∀	∀	∀

78. How often do you experience the reactions that are described below?
(Put an X for each line)

	Never	Seldom	Some-times	Often	Always
* I feel anxious and don't know what to do in an embarrassing situation ………………………	∀	∀	∀	∀	∀
* I feel anxious when I am with others and have to do something while they watch me do it *(ex: be in a play, play music, sports)* ……………………..	∀	∀	∀	∀	∀
* I feel anxious when I have to speak or read aloud in front of a group of people …………………	∀	∀	∀	∀	∀
* Before I go someplace where I'm going to be with people (*ex: a party, school, football game*) I sweat, my heart beats fast and/or I get a headache or stomach ache …………………	∀	∀	∀	∀	∀
* Before I go to a party or someplace with other people I think about what could go wrong *(ex: that I make mistakes, seem dumb and/or…what if they see how frightened I am!)* ………	∀	∀	∀	∀	∀
* I feel anxious and don't know what to do when I'm in a new situation ………………………………	∀	∀	∀	∀	∀

79. How have you thought and felt about yourself and about your family in <u>the past month</u>?
(Put an X for each line)

	Totally agree	Agree	Average	Disagree	Totally disagree
* I easily make others feel comfortable around me ………..	∀	∀	∀	∀	∀
* In my family we share views of what is important in life....	∀	∀	∀	∀	∀
* I easily find new friends ……………………………………	∀	∀	∀	∀	∀
* I feel comfortable with my family …………………………..	∀	∀	∀	∀	∀
* I am good at talking to new people …………………..…….	∀	∀	∀	∀	∀
* My family view the future as positive, even when very sad things happen…………………………..	∀	∀	∀	∀	∀
* I always find something fun to talk about…………………..	∀	∀	∀	∀	∀
* In my family we support each other… ……………………	∀	∀	∀	∀	∀

81. Have you during <u>the past month</u>:
(Put an X for each line)

	Almost every night	Often	Some-times	Never
* Had difficulty falling asleep in the evening	∀	∀	∀	∀
* Woke too early and couldn't fall asleep again	∀	∀	∀	∀

82. Have any of the following things happened to you? *(Put an X for each line)*

	No	Yes, last year	Yes, in my life
* That someone in your family has been seriously ill…………..	∀	∀	∀
* Death of a loved one……….. ……………………………..	∀	∀	∀
* A catastrophe (fire, avalanche, tidal wave, hurricane, etc.)……	∀	∀	∀
* A serious accident (ex: a very serious car accident) …………..	∀	∀	∀
* Been violently hurt (beaten or injured) …………………….	∀	∀	∀
* Seen others violently hurt …………………………………….	∀	∀	∀
* Been put in sexually uncomfortable/abusive situations by someone about your age ……………………………….	∀	∀	∀
* Been put in sexually uncomfortable/abusive situations by an adult…………………………………………………	∀	∀	∀
* Been threatened or physically harassed by other students at school for a long time……………………………	∀	∀	∀
* Received painful or frightening treatment at the hospital while being treated for an illness or injury…………………	∀	∀	∀
* Experienced something else that was very frightening, dangerous or violent………………………………………..	∀	∀	∀

IF YOU ANSWERED NO TO ALL THE ABOVE, SKIP TO QUESTION 86

If you have experienced any of the above in question 82:

83. **Do you still think very much about what happened?** Yes ∀ No ∀

If Yes, **do you have frightening thoughts, see images or hear sounds from the actual experience even when you don't want to?** Yes ∀ No ∀

84. When something reminds you about what happened do you become distant, afraid or sad?

Yes ∀ No ∀

85. Do you try to avoid talking about it, thinking about it or feel any feelings about what happened?

Yes ∀ No ∀

86. **If it was an injury or accident, do you have physical (bodily) late complications/problems from this?** Yes ∀ No ∀

LEISURE TIME

87. **How many teams or clubs are you part of? (for example: sports team, boy/girl scouts, band, etc.)**

None ∀ One ∀ Two or more ∀

88. **How often have you done any of these activities in <u>the past week</u>?**
(Put an X for each line)

	None	Once	2-3 times	4 times or more
* Visited someone you know……………………………………	∀	∀	∀	∀
* Was visited at home……………………………………………….	∀	∀	∀	∀
* Read a book, magazine, comic book…………………………….	∀	∀	∀	∀
* Listened to music ………… ………………………………	∀	∀	∀	∀
* Played an instrument ………… ………………………………	∀	∀	∀	∀
* Was out with friends for more than two hours in a row………..	∀	∀	∀	∀
* Was at a meeting or training with a club/team……. ……………	∀	∀	∀	∀
* Did a hobby……………. ………………………………………	∀	∀	∀	∀
* Did homework for more than one hour………..……………….	∀	∀	∀	∀
* Watched TV/DVD ……………………………………………..	∀	∀	∀	∀
* Played a computer/TV game……………………………………..	∀	∀	∀	∀
* Played, chatted or surfed the internet …………………......……	∀	∀	∀	∀
* Was at the library…………………………………………......……	∀	∀	∀	∀
* Went to the movies……..…………………………………..…….	∀	∀	∀	∀
* Was at a cafe or a meeting place for people your age…………...	∀	∀	∀	∀
* Was in a play, theatre…………………………………......……	∀	∀	∀	∀
* Did photography/film..……………………………………......……	∀	∀	∀	∀
* Went to a concert..………………………………………......….	∀	∀	∀	∀
* Went to watch a sport event, game……………………..…………	∀	∀	∀	∀
* Sang in a chore ……….. ……………………………………….....	∀	∀	∀	∀

89. **If you normally do some of the below listed activities, how long do you usually do so each time?** (Put an X for each line)

	Less than ½ hour	½ -1 hour	More than 1 hour
* Watch TV/DVD ………………………………………………..	∀	∀	∀
* Play computer/TV games……………………………………...	∀	∀	∀
* Play, chat or surf the internet …………………........…………	∀	∀	∀
* Listen to music.………………………………………….....…	∀	∀	∀

90. Do you have a mobile phone? Yes ∀ No ∀

If Yes:

* How long do you usually talk on your mobile phone a day? ___________Number of minutes

* How many text/picture messages do you usually get a day? ___________ Number of messages

* How many text/picture messages do you send a day? ___________Number of messages

FAMILY AND FRIENDS

91. About how many close friends do you have? (Include those you can speak confidentially with and who help you when you need help. Do not include people you live with, but other relatives should be included.) (One X)

None ∀ One ∀ Two or more ∀

92. Do you have a steady boyfriend/girlfriend? Yes ∀ No, not now, but before ∀ No ∀

93. Are your parents separated or divorced, or have they lived separately for more than one year? *(X the appropriate box and write in your age where necessary)*

∀ No

∀ Yes, they lived separately or were separated when I was _____ years old, but they later moved back together again.

∀ Yes, they were divorced or separated when I was ____ years old.

94. How well off do you think your family is compared to most others? (One X)

About the same as most others ∀ Better financial situation ∀ Worse financial situation ∀

95. Has there been or is there much arguing in your family? (One X)

No ∀ Yes, the past 12 months ∀ Yes, previously ∀

96. **How good is the relationship you have with your immediate family?** (Put an X for each line of the family members you have. If you have more than one sibling, think about the sibling you have the best relationship to.)

	Very good	Good	Not so good	Bad	*
Mother …………………………………………	∀	∀	∀	∀	
* Father …………………………………………	∀	∀	∀	∀	
* Sibling ……………………………………….	∀	∀	∀	∀	
* Stepmother or stepfather………………………	∀	∀	∀	∀	

97. **Do you often feel lonely?** (One X)

* Very often …………. ∀
* Often …………………..∀
* Sometimes ……………∀
* Seldom …………………………………..∀
* Very seldom or never……………….. ∀

SCHOOL

98. Do any of the following things happen to you at school, or have any of them happened?
(Put an X for each line)

	Never	Some-times	Often	Very often
* Have difficulties concentrating during class	∀	∀	∀	∀
* Think that gym or art is fun	∀	∀	∀	∀
* Think other classes are fun	∀	∀	∀	∀
* Argue with the teacher	∀	∀	∀	∀
* Look forward to going to school	∀	∀	∀	∀
* Skip school	∀	∀	∀	∀
* Understand what is being taught	∀	∀	∀	∀
* Have fun during recess/break time	∀	∀	∀	∀
* Are satisfied with your test results	∀	∀	∀	∀
* Have fistfights	∀	∀	∀	∀
* Are reprimanded by the teacher	∀	∀	∀	∀
* Cannot manage to be calm/sit still during class	∀	∀	∀	∀
* Become bored or dissatisfied	∀	∀	∀	∀
* Receive help for reading or writing problems	∀	∀	∀	∀
* Are called a negative name by students for a long time	∀	∀	∀	∀
*****Are snubbed/excluded by the students for a long time	∀	∀	∀	∀

HEALTH SERVICES

99. During <u>the last 12 months</u> have you been to: (Put an X for each line)

	Yes	No
* General practitioner (family doctor, doctor outside the hospital)…………………	∀	∀
* Doctor at the hospital ………………………………………………………………	∀	∀
* Child health care clinic run by nurses………………………………………………..	∀	∀
* School health services …………………………………………………………….	∀	∀
* Psychologist …………………………………………………………………………	∀	∀
* Physiotherapist ……………………………………………………………………….	∀	∀
* Chiropractor …………………………………………………………………………	∀	∀
* Other practitioner (naturopath, reflexologist, laying on of hands, healer, psychic, etc.)……………………………………………	∀	∀

100. Have you been admitted to the hospital <u>during the past 12 months</u>?

Yes ∀ No ∀

101. How often have you been absent from school due to illness <u>during the last 12 months</u>?

Less than 1 week ∀ 1-2 weeks ∀ More than 2 weeks ∀

PHYSICAL DEVELOPMENT

Below are some questions about physical changes that occur through adolescence.

102. During the teenage years there are periods where one grows quickly (growing spurt). Have you noticed that your body has grown quickly (become taller)? (One X)

* No, I have not begun to grow ………………………………………………………… ∀
* Yes, I have barely begun a growing spurt ……………………………………………… ∀
* Yes, I've clearly begun a growing spurt ………………………………………………… ∀
* Yes, it seems that I'm finished with growing spurts ………………………………… ∀

103. Concerning hair on your body (under your arms and your crotch/groin)? Would you say that the hair on your body has: (One X)

* Not begun to grow yet …………………………………………………………………. ∀
* Barely begun to grow ……………………………………………………………………. ∀
* Quite clearly begun to grow ……………………………………………………………. ∀
* It seems that my body hair has grown in …………………………………………… ∀

104. When you look at yourself, do you think that you are physically maturing/have physically matured earlier or later than others your own age? (One X)

* Much earlier …………………. ∀
* Earlier ……. …………………. ∀
* A little bit earlier…………….. ∀
* The same as others ………… ∀
* A little bit later ………………………… ∀
* Later ……………..……………………... ∀
* Much later ……………..……………….. ∀

QUESTIONS FOR BOYS

105. Has your voice begun to change? (One X)

* No, hasn't begun yet ……………………………………………………………………. ∀
* Yes, has just barely begun ……………………………………………………………………… ∀
* Yes, has clearly begun …………………………………………………………………... ∀
* It seems my voice has finished changing …………………………………………….. ∀

106. Has facial hair begun to grow (moustache or beard)? (One X)

* No, hasn't begun yet ……………………………………………………………………. ∀
* Yes, has just barely begun ……………………………………………………………………… ∀
* Yes, has clearly begun ……………………………………………………………………. ∀
* Yes, I have quite a lot of facial hair …………………………………………………… ∀

QUESTIONS FOR GIRLS

107. Have you begun to develop breasts? (One X)

* No, haven't begun yet ………..∀ * Yes, have quite clearly begun …………………….. ∀
* Yes, have barely begun ………..∀ * It seems my breasts are fully developed …..…. ∀

108. Have you begun menstruating (gotten your period)? Yes ∀ No ∀

IF YOU ANSWERED "NO", GO TO PAGE 22

109. How old were you when you first began menstruating?

I was ________years old and ________months.

110. How many times have you menstruated in the last 12 months? ___________ times

111. How long is it usually between your menstruation periods? (From the first day of a period to the first day of the next period)

Less than 3 weeks ∀ 3-4 weeks ∀ More than 4 weeks ∀

112. Have you ever missed (not gotten) your period for several months after a regular period? (without being pregnant)? (One X)

*Yes, 2-5 mos. …………………..∀ * Yes, more than 1 year …………………………………. ∀
*Yes, 6-12 mos. …………………∀ * No, never ……….…………………….…………………….. ∀

113. Have you ever taken birth control pills or the mini-pill?
Yes, I take them now ∀ Yes, I took them before ∀ No ∀

If Yes:

How old were you when you first began taking birth control pills/mini-pills? ______ years old

How long in total have you taken birth control pills/mini-pills? _______ years old

FOR STUDENTS IN HIGH SCHOOL

These questions are only to be answered by High School students.

114. During the last year, have you often felt that you pressured yourself or continuously pushed yourself?

Yes ∀ No ∀ Don't know ∀

115. Do you feel that you are constantly short of time, even in your everyday tasks?

* Always, or almost always ………………………… ∀
* Sometimes …………………………………… ∀
* Never…………………………………………… ∀

116. Have you ever had thoughts about taking your own life? Yes ☐ No ☐

117. Have you ever used anabolic steroids or other performance enhancing drugs?

Yes ☐ No ☐

118. Have you ever had sexual intercourse? Yes ∀ No ∀

If Yes, **How old were you the first time?** _______ years old

119. For GIRLS: Have you ever become pregnant when you did not want to be?

Yes ∀ No ∀

120. For BOYS: Have you ever gotten a girl pregnant without intending to?

Yes ∀ No ∀ Don't know ∀

For BOTH boys and girls:
If Yes,

How old were you when this happened? ____ years old

Was the result an abortion? Yes ∀ No ∀ Don't know ∀

COMMENTS

If you have time, you could write here about what you think is important, but was not asked about in this questionnaire. What are your thoughts about being young these days? What do feel can be improved upon concerning health and wellbeing for youth of today?

Thank you for your contribution ☺

Sincerely,

Turid Lingaas Holmen, førsteamanuensis/barnelege
Ung-HUNT leder

HUNT forskningssenter, Neptunveien 1, 7650 Verdal
Telefon: 74075180
