# Supplementary material for: Risk factors and risk profiles for neck pain in young adults: Prospective analyses from adolescence to young adulthood—The North-Trøndelag Health Study
Source: PLoS One. 2021 Aug 12;16(8):e0256006. doi: 10.1371/journal.pone.0256006 (PMC8360564; doi:10.1371/journal.pone.0256006)
Supplement: Attachment — Submitted filename: Response to reviewers.docx [file pone.0256006.s009.docx]

**Response to reviewers**

Reviewer #1: (No Response)

Reviewer #2: (No Response)

Reviewer #3: Authors responded adequately to all reviewers suggestions and made essential improvements with their revised manuscript. Some minor changes should be made as follows

**Author comment:** Thank you for the constructive comments. The suggested changes are conducted as described below.

1) Line 52 Please provide a reference for the following statement if exists ‘The high prevalence of neck pain in adolescents …’

**Author comment:** Thank you for this comment, we agree that this statement should be supported by references.

**Author action**:

Page 1, Line 52: We provided two references to support the statement that neck pain is prevalent in adolescents.

2) Line 224-228 Please provide a reference to justify the formula used

**Author comment:** Thank you for pointing this out.

**Author action**:

Page 8, Line 225: We inserted a references to justify the formula used.

3) Line 326-330 Please specify that your findings are describing an association in adolescents in Norway

**Author comment**: Thank you for highlighting this, we rewrote the sentence.

**Author changes**:

Page 13, Line 327: *This study found that female sex, low level of physical activity, loneliness, headache/migraine, back pain, and neck/shoulder pain in adolescence were risk factors for having neck pain in young adulthood in Norwegian adolescents.*

4) Line 355-356 Similar as above comment

**Author comment:** Thank you for highlighting this, we agree that this should be specified.

**Author actions:**

Page 15, Line 357: We rewrote the sentence: *Our finding that headache/migraine, neck/shoulder, and back pain in adolescence were associated with neck pain in young adulthood among Norwegian adolescents is supported in the literature*
